# Advisory Committee on Immunization Practices Recommended Immunization Schedule for Children and Adolescents Aged 18 Years or Younger — United States, 2017

**DOI:** 10.15585/mmwr.mm6605e1

**Published:** 2017-02-10

**Authors:** Candice L. Robinson, José R. Romero, Allison Kempe, Cynthia Pellegrini, Advisory Committee on Immunization Practices (ACIP) Child/Adolescent Immunization Work Group

**Affiliations:** ^1^Immunization Services Division, National Center for Immunization and Respiratory Diseases, CDC; ^2^University of Arkansas for Medical Sciences and Arkansas Children's Hospital, Little Rock, Arkansas; ^3^Department of Pediatrics, University of Colorado Anschutz Medical Campus, Denver, Colorado; ^4^March of Dimes, Washington, DC.

In October 2016, the Advisory Committee on Immunization Practices (ACIP) approved the Recommended Immunization Schedule for Children and Adolescents Aged 18 Years or Younger—United States, 2017. The 2017 child and adolescent immunization schedule summarizes ACIP recommendations, including several changes from the 2016 immunization schedules, in three figures, and footnotes for the figures. These documents can be found on the CDC immunization schedule website (https://www.cdc.gov/vaccines/schedules/index.html). These immunization schedules are approved by ACIP (https://www.cdc.gov/vaccines/acip/index.html), the American Academy of Pediatrics (https://www.aap.org), the American Academy of Family Physicians (https://www.aafp.org), and the American College of Obstetricians and Gynecologists (http://www.acog.org). Health care providers are advised to use the figures and the combined footnotes together. The full ACIP recommendations for each vaccine, including contraindications and precautions, can be found at https://www.cdc.gov/vaccines/hcp/acip-recs/index.html. Providers should be aware that changes in recommendations for specific vaccines can occur between annual updates to the childhood/adolescent immunization schedules. If errors or omissions are discovered within the child and adolescent schedule, CDC posts revised versions on the CDC immunization schedule website.*

Printable versions of the 2017 immunization schedules for children and adolescents aged 18 years or younger also are available at the website and ordering instructions for laminated versions and easy-to-read versions for parents also are available at the immunization schedule website.

For further guidance on the use of each vaccine included in the schedules, including contraindications and precautions, health care providers are referred to the respective ACIP vaccine recommendations at https://www.cdc.gov/vaccines/hcp/acip-recs/index.html.

## Changes in the 2017 Child and Adolescent Immunization Schedule

Changes in the 2017 immunization schedules for children and adolescents aged 18 years or younger include new or revised ACIP recommendations for influenza (*1*); human papillomavirus (*2*); hepatitis B (*3*); *Haemophilus influenzae* type B (*4*); pneumococcal; meningococcal (*5*,*6*); and diphtheria and tetanus toxoids and acellular pertussis (*7*) vaccines.

**Figure 1**. Changes to the 2017 figure from the 2016 schedule^†^ are as follows:

- The 16-year age column has been separated from the 17–18-year age column to highlight the need for a meningococcal conjugate vaccine booster dose at age 16 years.
- Live attenuated influenza vaccine (LAIV) has been removed from the influenza row.
- A blue bar was added for human papillomavirus vaccine (HPV) for children aged 9–10 years, indicating that persons in this age group may be vaccinated (even in the absence of a high-risk condition).

**Figure 3.** A new figure, “Figure 3. Vaccines that might be indicated for children and adolescents aged 18 years or younger based on medical indications,” has been added. The purpose of this figure is to do the following:

- Demonstrate most children with medical conditions can (and should) be vaccinated according to the routine child/adolescent immunization schedule.
- Indicate when a medical condition is a precaution or contraindication to vaccination.
- Indicate when additional doses of vaccines may be necessary because of a child’s or adolescent’s medical condition. Providers should consult the relevant footnotes for additional information.

**Footnotes.** Changes to the footnotes for the figures are as follows:

- The Hepatitis B vaccine (HepB) footnote was revised to reflect that the birth dose of HepB should be administered within 24 hours of birth.
- The diphtheria and tetanus toxoids and acellular pertussis vaccine (DTaP) footnote was revised to more clearly present recommendations following an inadvertently early administered fourth dose of DTaP.
- Within the *Haemophilus influenzae* type b vaccine (Hib) footnote, Comvax was removed from the routine vaccination portion of footnote. This vaccine has been removed from the market, and all available doses have expired. Additionally, Hiberix has been added to the list of vaccines that may be used for the primary vaccination series.
- Within the pneumococcal vaccine footnote, references to 7-valent pneumococcal conjugate vaccine (PCV7) have been removed. All healthy children who might have received PCV7 as part of a primary series have now aged out of the recommendation for pneumococcal vaccine.
- The influenza vaccine footnote has been updated to indicate that LAIV should not be used during the 2016–2017 influenza season.
- The meningococcal vaccines footnote has been updated to include recommendations for meningococcal vaccination of children with human immunodeficiency virus (HIV) infection and to reflect recommendations for the use of a 2-dose Trumenba (meningococcal B vaccine) schedule.
- The tetanus and diphtheria toxoids and acellular pertussis vaccine (Tdap) footnote for vaccination of pregnant adolescents between gestational weeks 27–36 has been updated to reflect a preference for vaccination earlier during this period. Currently available data suggest that vaccinating earlier in the 27 through 36–week period will maximize passive antibody transfer to the infant.
- The footnote for HPV vaccine has been updated to include the new 2-dose schedule for persons initiating the HPV vaccination series before age 15 years. In addition, bivalent HPV vaccine has been removed from the schedule. This vaccine has been removed from the U.S. market, and all available vaccine doses have expired.

## Footnotes

* CDC encourages organizations that previously have relied on copying the schedules to their websites instead to use syndication as a more reliable method for displaying the most current and accurate immunization schedules on an organization's website. Use of content syndication requires a one-time step that ensures an organization's website displays current schedules as soon as they are published or revised; instructions for the syndication code are available on CDC’s website (https://www.cdc.gov/vaccines/schedules/syndicate.html). CDC also offers technical assistance for implementing this form of content syndication (e-mail request to ncirdwebteam@cdc.gov).

† Past immunization schedules are available at https://www.cdc.gov/vaccines/schedules/past.html.
